# Prevention of the Entrance of Air When Removing Fluid from the Pleuræ, Peritoneum, and Cavities of Abscesses

**Published:** 1850-04

**Authors:** 


*Prevention of the entrance of Air when removing Fluid from the Pleuræ, Peritoneum, and Cavities of Abscesses.*—At a meeting of the

Societé de Chirurgie, of Paris, held on the 14th of November, a letter was read from M. Raciborski, of which the Union Médicale, for November, gives the following extract.

A wet, collapsed hog's bladder is fixed to the outlet of the canula which is to be introduced. When the trocar has sufficiently entered the cavity, the bladder must be supported by the left hand of the operator, the right being used to withdraw the trocar, and so allow the fluid to flow through the canula into the bladder. If the bladder be insufficient to contain the whole of the fluid to be withdrawn, the flow has to be stopped by pressing the side of the bladder against the outlet of the canula, whilst an assistant punctures the bladder in a convenient part, and thus evacuates its contents. By securing the opening by a ligature, the bladder may be made to serve for the evacuation of the whole of the fluid.—*Lond. Journ. of Med.*

